# Supplementary material for: Reference Values for Isometric, Dynamic, and Asymmetry Leg Extension Strength in Patients with Multiple Sclerosis
Source: Int J Environ Res Public Health. 2020 Nov 2;17(21):8083. doi: 10.3390/ijerph17218083 (PMC7662302; doi:10.3390/ijerph17218083)
Supplement: Supplementary file 1 [file ijerph-17-08083-s001.zip › Table S1 Force variables by type of Multiple Sclerosis and gender..pdf]

Table S1: Force variables by type of Multiple Sclerosis and gender.

Male

	PP (P)				RR (R)				SP (S)				
	Mean	SD	Max.	Min.	Mean	SD	Max.	Min.	Mean	SD	Max.	Min.	P
1RM Bil.	87.1	± 24.5	120.0	49.0	93.5	± 25,4^{S}	160.0	33.0	74.2	± 28.5	130.0	16.0	0.028
1RM Rigth	40.4	± 15.0	64.0	15.0	47.3	± 17.7	90.0	14.0	54.5	± 12.5	77.0	38.0	0.236
1RM Left	46.5	± 19.1	75.0	21.0	50.3	± 17.8	90.0	21.0	52.0	± 13.2	72.0	30.0	0.812
Isometric Bil.	94.1	± 24.4	141.2	56.7	105.4	± 31,9^{S}	182.6	46.0	82.8	± 31.3	144.7	24.8	0.006
Isometric Rigth	45.8	± 11.6	65.8	22.5	48.9	± 16.9	101.6	10.0	39.5	± 16.5	70.4	17.5	0.084
Isometric Left	49.8	± 14.1	66.4	25.7	49.9	± 17.9	108.6	9.0	39.3	± 17.0	71.0	4.5	0.057
Asymmetry 1RM	17.1	± 17.6	46.4	0.0	10.6	± 15	57.6	0.0	5.4	± 10.9	29.0	0.0	0.328
Asymmetry MVIC	19.8	± 14.4	39.5	0.8	18.0	± 14.8^{S}	78.4	0.2	28.5	± 20.5	74.3	0.5	0.044

Female

	PP (P)				RR (R)				SP (S)				
	Mean	SD	Max.	Min.	Mean	SD	Max.	Min.	Mean	SD	Max.	Min.	P
1RM Bil.	51.6	± 18.3	83.0	14.0	61.9	± 21,7^{S}	120.0	6.0	48.8	± 19.9	95.0	12.0	0.014
1RM Rigth	22.0	± 7.9	35.0	13.0	29.8	± 11.4	59.0	8.0	24.9	± 4.4	35.0	18.0	0.086
1RM Left	27.9	± 8.8	37.0	16.0	30.6	± 11,3^{S}	55.0	0.0	21.7	± 8.3	36.0	10.0	0.037
Isometric Bil.	56.2	± 16.3	102.6	27.9	67.2	± 19^{S,P}	117.0	19.5	51.1	± 18.2	104.8	16.3	0.000
Isometric Rigth	26.2	± 7.4	37.9	13.5	33.3	± 9,9^{S,P}	62.1	10.0	25.9	± 10.2	47.1	5.4	0.000
Isometric Left	27.1	± 8.6	51.0	9.3	32.5	± 10,3^{S}	56.5	9.0	25.0	± 9.7	55.3	9.9	0.001
Asymmetry 1RM	21.5	± 19.6	55.6	0.0	9.7	± 16.4	74.2	0.0	6.8	± 10.8	27.8	0.0	0.137
Asymmetry MVIC	20.9	± 13.7	52.0	0.3	15.0	± 13.9^{S}	67.7	0.0	25.1	± 15.6	57.8	3.0	0.003

The strength is expressed in kilogram_force (Kg_f). MVIC= maximal voluntary isometric contraction; 1RM= one repetition maximum; Bil.= bilateral; asymmetry is indicated in %; SD = standard deviation; Max = maximum value; Min = minimum value. PP = Primary-Progressive; RR = Relapsing–Remitting; SP = Secondary-Progressive. Letters (P, R, S) indicate significant differences between groups. asymmetry is indicated in percentage values.
