# Supplementary material for: Reference Values for Isometric, Dynamic, and Asymmetry Leg Extension Strength in Patients with Multiple Sclerosis
Source: Int J Environ Res Public Health. 2020 Nov 2;17(21):8083. doi: 10.3390/ijerph17218083 (PMC7662302; doi:10.3390/ijerph17218083)
Supplement: Supplementary file 1 [file ijerph-17-08083-s001.zip › Table S2 Force variables by neurological disability level and gender..pdf]

Table S2*: Force variables by neurological disability level and gender.*

Male

	Mild (L)				Moderate (M)				Severe (S)				
	Mean	SD	Max.	Min.	Mean	SD	Max.	Min.	Mean	SD	Max.	Min.	P
1RM Bil.	98.9	± 17.7 ^{S}	130	67	87.6	± 27.9	160	49	74.4	± 32.5	150	16	0.015
1RM Rigth	51.9	± 15	77	26	47.9	± 18.6	90	15	37.1	± 15.8	55	14	0.128
1RM Left	55.3	± 13 ^{S}	85	36	52.2	± 20.0	90	28	33.7	± 13.7	55	21	0.016
IMVIC Bil.	115	± 27	179	76.1	101.1	± 29.4	176.1	56.7	7.8	± 28.7 ^{L,M}	161.3	24.8	0.000
MVIC Rigth	54.2	± 15	102	26.2	47	± 13.3	87	22.1	35.4	± 15.3 ^{L,M}	65.5	10	0.000
MVIC Left	54.6	± 13	81.6	30.5	48.9	± 16.9	89.1	25	35.6	± 14.1 ^{L,M}	64.7	4.5	0.000
Asymmetry 1RM	11.5	± 12.3	37.3	0	12.6	± 16.1	46.4	0	8.7	± 21.6	57.6	0	0.875
Asymmetry MVIC	17.9	± 11.0	41.8	0.7	17.8	± 13.2	44.6	0.5	28.1	± 22.9	74.3	0.2	0.050

Female

	Mild (L)				Moderate (M)				Severe (S)				
	Mean	SD	Max.	Min.	Mean	SD	Max.	Min.	Mean	SD	Max.	Min.	P
1RM Bil.	67.3	± 19.4 ^{M,S}	115	23	54.5	± 17.3	110	15	46	± 23.1	95	6	0.000
1RM Rigth	31.6	± 9.9 ^{S}	59	18	26.3	± 10.9	50	8	22	± 6	28	13	0.019
1RM Left	31.9	± 12	55	0.0	25.7	± 9.9	45	10	25.3	± 7.1	36	17	0.044
IMVIC Bil.	74.5	± 17.4 ^{M,S}	117	40	56.7	± 14.1	94.7	16.3	49	± 19.4	104.8	19.2	0.000
MVIC Rigth	36.3	± 8.2 ^{M,S}	62.1	22.4	27.9	± 7.4	45.8	11.1	23.5	± 10.5	47.1	5.4	0.000
MVIC Left	35.3	± 9.5 ^{M,S}	56.5	12	27.5	± 8.6	51	10	23.9	± 10.8	55.3	9	0.000
Asymmetry 1RM	7.1	± 9.7	34.0	0.0	10.3	± 20.3	74.2	0.0	18.3	± 15.9	36	0.0	0.184
Asymmetry MVIC	12.0	± 11.8 ^{M,S}	62.5	0.0	20.6	± 15.6	67.7	0.0	24.6	± 14.3	57.8	0.7	0.000

The strength is expressed in kilogram_force (Kg_f). MVIC= maximal voluntary isometric contraction; 1RM= one repetition maximum; Bil.= bilateral; asymmetry is indicated in %; SD = standard deviation; Max = maximum value; Min = minimum value. Letters (L, M, S) indicate significant differences between groups. asymmetry is indicated in percentage values.
