# Supplementary material for: Reference Values for Isometric, Dynamic, and Asymmetry Leg Extension Strength in Patients with Multiple Sclerosis
Source: Int J Environ Res Public Health. 2020 Nov 2;17(21):8083. doi: 10.3390/ijerph17218083 (PMC7662302; doi:10.3390/ijerph17218083)
Supplement: Supplementary file 1 [file ijerph-17-08083-s001.zip › Table S3 and Table S4 Quartiles of force variables of sample by type of Multiple Sclerosis and gender_ And by EDSS degree and gender.pdf]

Table S3: Quartiles of force variables of sample by type of Multiple Sclerosis and gender.

	Male									Female								
	PP			RR			SP			PP			RR			SP		
	**Q1**	**Q2**	**Q3**	**Q1**	**Q2**	**Q3**	**Q1**	**Q2**	**Q3**	**Q1**	**Q2**	**Q3**	**Q1**	**Q2**	**Q3**	**Q1**	**Q2**	**Q3**
1RM Bil.	63.0	87.5	113.0	83.2	90.0	114.0	53.5	75.0	97.5	42.0	55.0	67.0	47.3	59.5	75.0	42.3	47.0	63.2
1RM Rigth	31.0	41.0	52.0	33.0	48.0	58.2	45.5	50.0	62.0	16.0	20.0	27.0	21.0	28.0	38.0	22.0	25.0	26.0
1RM Left	28.7	47.5	64.0	35.2	49.5	60.2	46.0	50.0	62.0	17.0	29.0	36.0	22.0	29.0	38.0	16.7	20.0	29.5
IMVIC Bil.	75.4	96.0	114.0	86.4	100.5	128.0	54.6	82.0	108.2	46.0	55.6	65.1	53.7	66.1	77.3	40.0	50.2	64.7
MVIC Rigth	41.0	45.0	51.4	39.5	46.7	57.9	24.4	37.3	51.1	18.6	27.3	32.2	27.2	31.9	39.7	20.0	28.0	33.0
MVIC Left	37.3	54.0	61.0	35.1	49.1	60.9	27.4	39.3	51.8	21.7	27.1	30.5	26.0	31.5	39.7	17.8	24.0	31.1
Asymmetry 1RM	0.0	16	29.1	0.0	1.8	17.9	0.0	3.9	9.1	5.4	22.9	36	0.0	0.0	10.8	0.0	8.3	14.7
Asymmetry MVIC	2.3	24.2	31.8	7.1	14.9	25	11.8	26.6	38.5	10	22.8	31.1	4.9	10.2	20.9	11.6	26.2	35.1

Table S4: Quartiles of force variables of sample by EDSS degree and gender.

	Male									Female								
	Mild			Moderate			Severe			Mild			Moderate			Severe		
	**Q1**	**Q2**	**Q3**	**Q1**	**Q2**	**Q3**	**Q1**	**Q2**	**Q3**	**Q1**	**Q2**	**Q3**	**Q1**	**Q2**	**Q3**	**Q1**	**Q2**	**Q3**
1RM Bil.	85.0	90.0	115.5	66.5	82.0	112.5	51.7	75.5	92.5	55.5	64.0	80.0	43.0	50.0	67.0	28.0	47.0	61.0
1RM Rigth	42.2	52.5	66.2	37.2	47.0	58.0	22.7	38.0	52.2	25.0	28.0	38.0	19.0	21.5	35.0	16.0	25.0	26.7
1RM Left	45.2	53.5	66.2	30.2	52.5	65.7	21.0	31.0	50.0	25.0	32.0	37.7	18.7	23.5	33.5	18.7	25.0	32.5
IMVIC Bil.	94.5	109.4	129.0	74.0	102.5	128.8	55.5	80.1	95.5	65.0	72.0	86.7	45.9	56.4	66.3	32.7	50.3	61.1
MVIC Rigth	42.2	51.0	66.0	39.0	46.2	53.7	23.5	36.0	44.0	30.4	34.5	42.6	23.0	28.4	32.0	14.6	21.5	29.3
MVIC Left	46.5	52.8	61.0	33.3	50.0	62.4	27.9	38.7	42.9	28.1	33.0	42.3	20.6	26.7	32.9	24.3	25.8	29.3
Asymmetry 1RM	0.0	9.8	21.2	0.0	4.6	26.5	0	1.5	3.6	0.0	4.8	10.7	0.0	5.2	9.1	0.0	23.2	31.9
Asymmetry MVIC	8.8	16.8	26.2	5.4	15.5	28.1	7.8	25.1	44.4	4.4	7.3	18.8	7.8	17	30.6	14.8	25	32.3

The strength is expressed in kilogram_force (Kg_f). MVIC= maximal voluntary isometric contraction; 1RM= one repetition maximum; Bil.= bilateral; asymmetry is indicated in %; SD = standard deviation; Max = maximum value; Min = minimum value. PP = Primary-Progressive; RR = Relapsing–Remitting; SP = Secondary-Progressive. Asymmetry is indicated in percentage values.
